# AIDS incidence trends at presentation and during follow-up among HIV-at-risk populations: a 15-year nationwide cohort study in Taiwan

**DOI:** 10.1186/s12889-018-5500-z

**Published:** 2018-05-03

**Authors:** Chun-Yuan Lee, I-An Jen, Yu-Ching Lan, Yung-Feng Yen, Pei-Hung Chuang, Marcelo Chen, Yun Lee, Yi-Ming A. Chen

**Affiliations:** 1 Division of Infectious Diseases, Department of Internal Medicine, Kaohsiung Medical University Hospital, Kaohsiung Medical University, Kaohsiung, Taiwan; 2 Graduate Institute of Medicine, Kaohsiung Medical University, Kaohsiung, Taiwan; 3 Center for Infectious Disease and Cancer Research (CICAR), Kaohsiung Medical University, No.100, Tzyou 1st Road, Kaohsiung, 807 Taiwan; 4 Department and Institute of Public Health, National Yang-Ming University, Taipei, Taiwan; 5 Department of Health Risk Management, China Medical University, Taichung, Taiwan; 6 Section of Infectious Diseases, Taipei City Hospital, Taipei, Taiwan; 7 School of Medicine, National Yang-Ming University, Taipei, Taiwan; 8 Department of Health and Welfare, College of City Management, University of Taipei, Taipei, Taiwan; 9 Center for Prevention and Treatment of Occupational Injury and Diseases, Taipei Veterans General Hospital, Taipei, Taiwan; 10 Division of Clinical Toxicology and Occupational Medicine, Department of Medicine, Taipei Veterans General Hospital, Taipei, Taiwan; 11 Department of Urology, Mackay Memorial Hospital, Taipei, Taiwan; 12 Department of Cosmetic Applications and Management, Mackay Junior College of Medicine, Nursing and Management, Taipei, Taiwan; 13 Department of Microbiology and Institute of Medical Research, College of Medicine, Kaohsiung Medical University, Kaohsiung, Taiwan

**Keywords:** Acquired immunodeficiency syndrome, Antiretroviral therapy, HIV, Homosexual, Heterosexual

## Abstract

**Background:**

Although Taiwan has implemented several important interventions for various HIV-at-risk populations to combat the HIV epidemic, little is known regarding AIDS incidence at presentation and during follow-up among the various HIV-at-risk populations in Taiwan. A better understanding of AIDS incidence trends would help improve patient care and optimize public health strategies aimed at further decreasing HIV-related morbidity and mortality.

**Methods:**

Data from Taiwan Centers for Disease Control-operated Notifiable Diseases Surveillance System and Taiwan National Health Insurance Research Database (1998–2012) was divided into five cohort periods (consecutive 3-year groups). Logistic regression was employed to identify factors associated with AIDS incidence at presentation. Time-dependent Cox regression was used to identify factors associated with AIDS incidence during the follow-up period.

**Results:**

Of 22,665 patients [mean age: 32 years; male (93.03%)], 6210 (27.4%) had AIDS incidence over 2 (1.16) [median (interquartile range)] years of follow-up. AIDS developed in ≤3 months of HIV diagnosis in 73.6% AIDS patients. AIDS incidence trends at presentation and during follow-up differed according to HIV transmission routes over the five periods: AIDS at presentation increased in the sexual contact groups (*P* < 0.001 for homosexuals/heterosexuals; 0.648 for bisexuals) but decreased to a nadir in period 3 and then increased slightly in period 5 (*P* < 0.001) in people who injected drugs (PWIDs). AIDS incidence during the follow-up period increased from period 1 to a peak in period 3 or 4, before declining slightly in period 5, in the sexual contact groups (*P* < 0.001 for homosexuals/heterosexuals; 0.549 for bisexuals). However, it increased throughout the five periods in PWIDs (*P* < 0.001). Older age, sexual contact group versus PWIDs, high versus low income level, cohort periods, and HIV diagnosis regions helped predict AIDS at presentation and during follow-up.

**Conclusions:**

Disparities in AIDS incidence trends in various HIV-at-risk populations reflect different sociodemographic variables of HIV exposure and the adopted HIV prevention strategies. This study suggests the urgent need for tailored strategies aimed at specific populations at presentation and during follow-up.

## Background

HIV-related mortality and morbidity rates have substantially decreased because of global efforts to improve access to antiretroviral therapy [1, 2]; however, high acquired immunodeficiency syndrome (AIDS) incidence, especially during the early phase of the HIV care, remains challenging. Although global campaigns encourage earlier HIV testing, substantial numbers of HIV-infected individuals do not enter healthcare systems until later stages, even when AIDS-defining events occur [3–5].

AIDS incidence is associated with higher HIV-related morbidity and mortality rates [5–8], higher HIV transmission rates to sexual partners [9, 10], increase health expenses [7, 11], and impaired immunological response to highly active antiretroviral therapy (HAART) [12]. Therefore, ongoing surveillance to monitor AIDS incidence trends at presentation and during follow-up and the characterization of the associated risk factors is essential. AIDS incidence at presentation indicates late presentation of HIV [3–5], whereas AIDS incidence during follow-up is related to access to HAART, thresholds of initiation of HAART, regimens of HAART, and adherence to HIV care. Therefore, a better understanding of AIDS incidence trends at presentation and during follow-up would help improve patient care and optimize public health strategies aimed at further decreasing HIV-related morbidity and mortality.

Since the first HIV case in Taiwan was reported in 1984, a total of 33,423 cases were reported until the end of 2016. Of these, 15,418 patients (46.1%) developed full-blown AIDS [13]. Taiwan has implemented several important interventions for various HIV-at-risk populations to combat the HIV epidemic [13]. These include free HAART and anonymous voluntary counseling and testing (VCT) for HIV among at-risk populations since 1997, harm-reduction programs among intravenous drug users (PWIDs) since 2005, and an HIV case management program since 2007. However, little is known regarding AIDS incidence trends at presentation and during follow-up among the various HIV-at-risk populations in Taiwan [14–16].

In this nationwide cohort study, we employed data from the Taiwan Centers for Disease Control (TCDC)-operated Notifiable Diseases Surveillance System (NDSS) and Taiwan National Health Insurance Research Database (NHIRD) (reference period: 1998–2012) to analyze AIDS incidence trends at presentation (thus reflecting issues of late presentation of HIV) and during follow-up (thus reflecting issues of access to HAART, thresholds of initiation of HAART, regimens of HAART, and adherence to HIV care) among various HIV-at-risk populations over five cohort periods.

## Methods

### Data sources

Data were obtained from the TCDC-operated NDSS and NHIRD databases. The TCDC-operated NDSS provides a national web-based platform for reporting and monitoring several communicable diseases, including HIV and AIDS. Since 1984, both HIV infection and AIDS are notifiable diseases by law in Taiwan. Once HIV infection cases are confirmed on the basis of positive HIV-1 western blot or polymerase chain reaction analysis results, diagnosing healthcare providers are required to report newly confirmed HIV-infection cases to the TCDC-operated NDSS ≤24 h of diagnosis. According to the United States CDC 1993 AIDS case definition [17], the reporting of AIDS is also mandatory within 24 h. Patients’ information, including identification number, date of birth, sex, home address, HIV-transmission route, date of HIV diagnosis, and AIDS incidence must be reported to TCDC-operated NDSS by diagnosing healthcare providers.

Taiwan National Health Insurance is a mandatory universal health insurance program that has provided comprehensive medical care to > 99% Taiwanese citizens since 1995 [18]. NHIRD, a large-scale, public database derived from the national health insurance system, contains registration files and original claims data for reimbursement. Researchers can apply for access to associated data from TCDC-operated NDSS and NHIRD. The data is anonymized before release.

### Study design and setting

First, new-HIV-infection cases from January 1, 1998 to December 31, 2012 were directly selected from NDSS. The selected cases were then linked to the Taiwan NHIRD. Following exclusion criteria were used: age < 15 years; sex category: unknown; incomplete data. To analyze AIDS incidence trends, the enrolled patients were stratified according to HIV diagnosis date into five (3-year) cohort periods: period 1 (January 1, 1998–December 31, 2000); period 2 (January 1, 2001–December 31, 2003); period 3 (January 1, 2004–December 31, 2006); period 4 (January 1, 2007–December 31, 2009); and period 5 (January 1, 2010–December 31, 2012). Each enrolled HIV patient was followed up until 24 months from the date of reporting to NDSS, or AIDS incidence, or death, whichever occurred first. Next, the sociodemographic variables, HIV-related variables, and associated outcomes were compared between the cohort periods. Finally, AIDS incidence trends at presentation and during the follow-up periods among various HIV-at-risk populations and their associated factors were analyzed.

### Variable collection and definitions

The control variables were sociodemographic variables, comorbidities, HIV diagnosis region, and cohort periods. Urbanization was categorized as residence in an urban or rural area [19]. Insurer income level was categorized into three levels: low (≤19,200 New Taiwan Dollars [NTD]), intermediate (19,201–40,000 NTD), and high (≥40,000 NTD) according to the average monthly income of the insured person [19]. For insured patients aged between 15 to 20 years, who have no income, the insured income indicates their parents’ income. The comorbidities in the study population were defined by NHIRD, and a person was considered to have comorbidities when the comorbidities were diagnosed in an inpatient setting or at ≥3 outpatient visits before HIV diagnosis [19].

HAART initiation was defined as the first recorded date of prescription of ≥3 antiretroviral agents (≥2 classes) or a triple nucleoside/nucleotide reverse transcriptase inhibitor regimen.

AIDS incidence at presentation was defined as the frequency of patients who developed AIDS ≤3 months of HIV diagnosis [4]. AIDS incidence during follow-up period was defined as those who did not develop AIDS ≤3 months of HIV diagnosis, but developed AIDS thereafter during the follow-up period. HIV diagnosis region was defined as the area from which physicians reported HIV diagnosis to TCDC, and was categorized into one of six administrative areas according to the TCDC NDSS: Taipei area, northern Taiwan, central Taiwan, southern Taiwan, Kaoping area, and eastern Taiwan.

### Primary and secondary outcomes

The primary outcomes were trends of AIDS incidence at presentation and during the follow-up period among the targeted HIV-at-risk populations over the five cohort periods. The secondary outcomes were factors associated with AIDS incidence at presentation and during the follow-up period.

### Statistical analysis

The values of categorical variables among the five cohort periods were compared using the Pearson’s χ2 or Fisher’s exact test. The values of continuous variables were compared using the analysis of variance. The trend analyses of AIDS incidence at presentation and during the follow-up period through five follow-up periods among HIV-at-risk populations were performed using the Cochran–Armitage trend test with modified ridit scores.

Univariable analysis and multivariable logistic regression were employed to identify factors associated with AIDS incidence at presentation. All variables used in univariable analysis were selected for subsequent multivariable logistic regression.

The probability of AIDS-free survival stratified by the cohort period in different HIV-at-risk populations was estimated using the Kaplan–Meier survival curves and log-rank testing. Time-dependent Cox regression was used to identify factors associated with AIDS incidence during the follow-up period. In these models, HAART was regarded as a time-dependent covariable, whereas other confounders, such as sociodemographic variables (age, sex, urbanization, and income level); HIV transmission route; comorbidities; HIV diagnosis region; and cohort period—collected at baseline—were regarded as fixed covariates.

Further, 95% confidence intervals (CIs) of odds ratios (ORs) or hazard ratios (HRs) were calculated to estimate each variable’s effects and the direction of associations. All tests were two-tailed, and *P* < 0.05 was considered significant. All data management and analyses were performed using the SAS 9.4 software package (SAS Institute, Cary, NC, USA).

## Results

### Participant selection

**Fig. 1:**
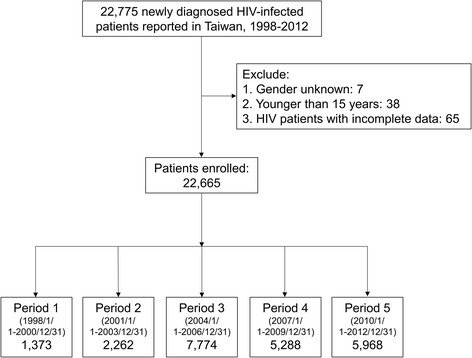
Schematic illustration of the study population

A total of 22,665 HIV patients were enrolled: 1373 from period 1; 2262 from period 2; 7774 from period 3; 5288 from period 4; and 5968 from period 5 (Fig. 1).

### Characteristics of study participants

Table 1 summarizes the baseline sociodemographic variables, comorbidities, HIV diagnosis region, and HIV-related outcomes for the five-period cohorts. Over a median (interquartile range, IQR) follow-up period of 2 (1.16) years, 93.0% patients were men, with a mean age at presentation of 32 (±10) years. The four major routes of HIV transmission were homosexual contact (43.2%), PWID (29.3%), heterosexual contact (18.3%), and bisexual contact (8.4%). Overall, gbMSM (gay, bisexual, and homosexuals) accounted for 51.6% of the HIV population. HIV epidemics in the various HIV transmission route groups differed by cohort period: PWID percentages increased from 1.1 to 62.9% between periods 1 and 3, and then declined to 5.3% in period 5; homosexual percentages declined to 19.7% in period 3, and then increased to 67.6% in period 5. In total, 6210 (27.4%) patients developed AIDS during five cohort periods; the lowest and the highest percentages were in period 3 (17.7%) and in period 5 (35.9%), respectively. Among the 6210 AIDS patients, 4571 (73.6%) had developed at presentation; the lowest and highest numbers of cases were in period 3 (59.4%) and period 1 (90.2%), respectively. Among the 6210 patients, 1639 (26.4%) developed AIDS during the follow-up period; the lowest and highest numbers of cases were in period 1 (9.8%) and period 3 (40.6%), respectively.

**Table 1:** Sociodemographic, HIV-related variables, and comorbidities in patients newly diagnosed with HIV infection

	Period 1	Period 2	Period 3	Period 4	Period 5	Total (*n* = 22,665)	*P*-value
	1/1/1998 to 12/31/2000 (*n* = 1373)	1/1/2001 to 12/31/2003 (*n* = 2262)	1/1/2004 to 12/31/2006 (*n* = 7774)	1/1/2007 to 12/31/2009 (*n* = 5288)	1/1/2010 to 12/31/2012 (*n* = 5968)		
Follow-up period, median (IQR; years)	2 (1.94)	2 (0.25)	2 (0)	2 (1.75)	2 (1.92)	2 (1.16)	< 0.001
Age, mean (IQR; years)	34.56 (11.77)	33.91 (11.66)	33.05 (9.36)	33.17 (10.44)	31.08 (10.26)	32.74 (10.31)	< 0.001
Age group, *n* (%)							< 0.001
≤ 30	610 (44.4)	1058 (46.8)	3620 (46.6)	2530 (47.8)	3487 (58.4)	11,305 (49.9)	
31–40	445 (32.4)	723 (32.0)	2650 (34.1)	1606 (30.4)	1541 (25.8)	6965 (30.7)	
41–50	165 (12.0)	260 (11.5)	1113 (14.3)	800 (15.1)	624 (10.5)	2962 (13.1)	
≥ 51	153 (11.1)	221 (9.8)	391 (5.0)	352 (6.7)	316 (5.3)	1433 (6.3)	
Sex, n (%)							< 0.001
Female	116 (8.5)	117 (5.2)	809 (10.4)	337 (6.4)	201 (3.4)	1580 (7.0)	
Male	1257 (91.5)	2145 (94.8)	6965 (89.6)	4951 (93.6)	5767 (96.6)	21,085 (93.0)	
Income level, *n* (%)							< 0.001
Low	805 (58.6)	1352 (59.8)	6147 (79.1)	2737 (51.8)	2744 (46.0)	13,785 (60.8)	
Intermediate	411 (29.9)	585 (25.8)	1109 (14.3)	1928 (36.5)	2388 (40.0)	6421 (28.3)	
High	157 (11.4)	325 (14.4)	518 (6.6)	623 (11.7)	836 (14.0)	2459 (10.9)	
Urbanization, *n* (%)							< 0.001
Rural	265 (19.3)	475 (21.0)	2933 (37.7)	1774 (33.6)	1937 (32.5)	7384 (32.6)	
Urban	1108 (80.7)	1787 (79.0)	4841 (62.3)	3514 (66.4)	4031 (67.5)	15,281 (67.4)	
HIV-transmission route, *n* (%)							< 0.001
Homosexual contact	574 (41.8)	1125 (49.7)	1532 (19.7)	2516 (47.6)	4032 (67.6)	9779 (43.2)	
Heterosexual contact	578 (42.1)	729 (32.2)	974 (12.5)	946 (17.9)	925 (15.5)	4152 (18.3)	
Bisexual contact	197 (14.4)	278 (12.3)	338 (4.4)	453 (8.6)	632 (10.6)	1898 (8.4)	
PWID	15 (1.1)	108 (4.8)	4887 (62.9)	1322 (25.0)	314 (5.3)	6646 (29.3)	
Other^a^	9 (0.6)	22 (1.0)	43 (0.5)	51 (1.0)	65 (1.1)	190 (0.8)	
Comorbidities, *n* (%)							
DM	16 (1.2)	60 (2.7)	193 (2.5)	144 (2.7)	155 (2.6)	568 (2.5)	< 0.05
CKD	13 (1.0)	25 (1.1)	94 (1.2)	84 (1.6)	91 (1.5)	307 (1.4)	0.114
CHF	4 (0.3)	9 (0.4)	23 (0.3)	30 (0.6)	26 (0.4)	92 (0.4)	0.177
COPD	24 (1.8)	74 (3.3)	173 (2.2)	128 (2.4)	108 (1.8)	507 (2.2)	< 0.05
Cancer	44 (3.2)	129 (5.7)	439 (5.7)	451 (8.5)	583 (9.8)	1646 (7.3)	< 0.001
CVD	12 (0.9)	31 (1.4)	83 (1.1)	74 (1.4)	57 (1.0)	257 (1.1)	0.121
HAART in each cohort period, *n* (%)	605 (44.1)	1273 (56.3)	1546 (19.9)	1237 (23.4)	2345 (39.3)	7006 (30.9)	< 0.001
AIDS incidence in each cohort period, *n* (%)	438 (31.9)	519 (22.9)	1378 (17.7)	1732 (32.8)	2143 (35.9)	6210 (27.4)	< 0.001
AIDS incidence at presentation, *n* (%)	395 (90.2)	451 (86.9)	819 (59.4)	1274 (73.6)	1632 (76.2)	4571 (73.6)	< 0.001
AIDS incidence during the follow-up period, *n* (%)	43 (9.8)	68 (13.1)	559 (40.6)	458 (26.4)	511 (23.8)	1639 (26.4)	< 0.001
HIV diagnosis region, *n* (%)							< 0.001
Taipei area	622 (45.3)	1061 (46.9)	2266 (29.2)	1923 (36.4)	2027 (34.0)	7899 (34.9)	
Northern Taiwan	161 (11.7)	262 (11.6)	1278 (16.4)	661 (12.5)	850 (14.2)	3212 (14.2)	
Central Taiwan	238 (17.3)	339 (15.0)	1539 (19.8)	913 (17.3)	1052 (17.6)	4081 (18.0)	
Southern Taiwan	12 (0.9)	244 (10.8)	1057 (13.6)	593 (11.2)	669 (11.2)	2687 (11.9)	
Kaoping area	192 (14.0)	307 (13.6)	1535 (19.8)	1086 (20.5)	1191 (20.0)	4311 (19.0)	
Eastern Taiwan	36 (2.6)	49 (2.2)	99 (1.3)	112 (2.1)	179 (3.0)	475 (2.1)	

*Abbreviations*: *AIDS* acquired immunodeficiency syndrome, *COPD* chronic obstructive pulmonary disease, *CHF* chronic heart failure, *CKD* chronic kidney disease, *CVD* cardiovascular disease, *DM* diabetes mellitus, *HAART* highly active antiretroviral therapy, *IQR* interquartile range, *PWID* people who injected drugs, *SD* standard deviation

^a^Includes those exposed to blood products, mother-to-child transmission, and unknown exposures

### AIDS incidence trends at presentation and during the follow-up period according to HIV-transmission route

**Fig. 2:**
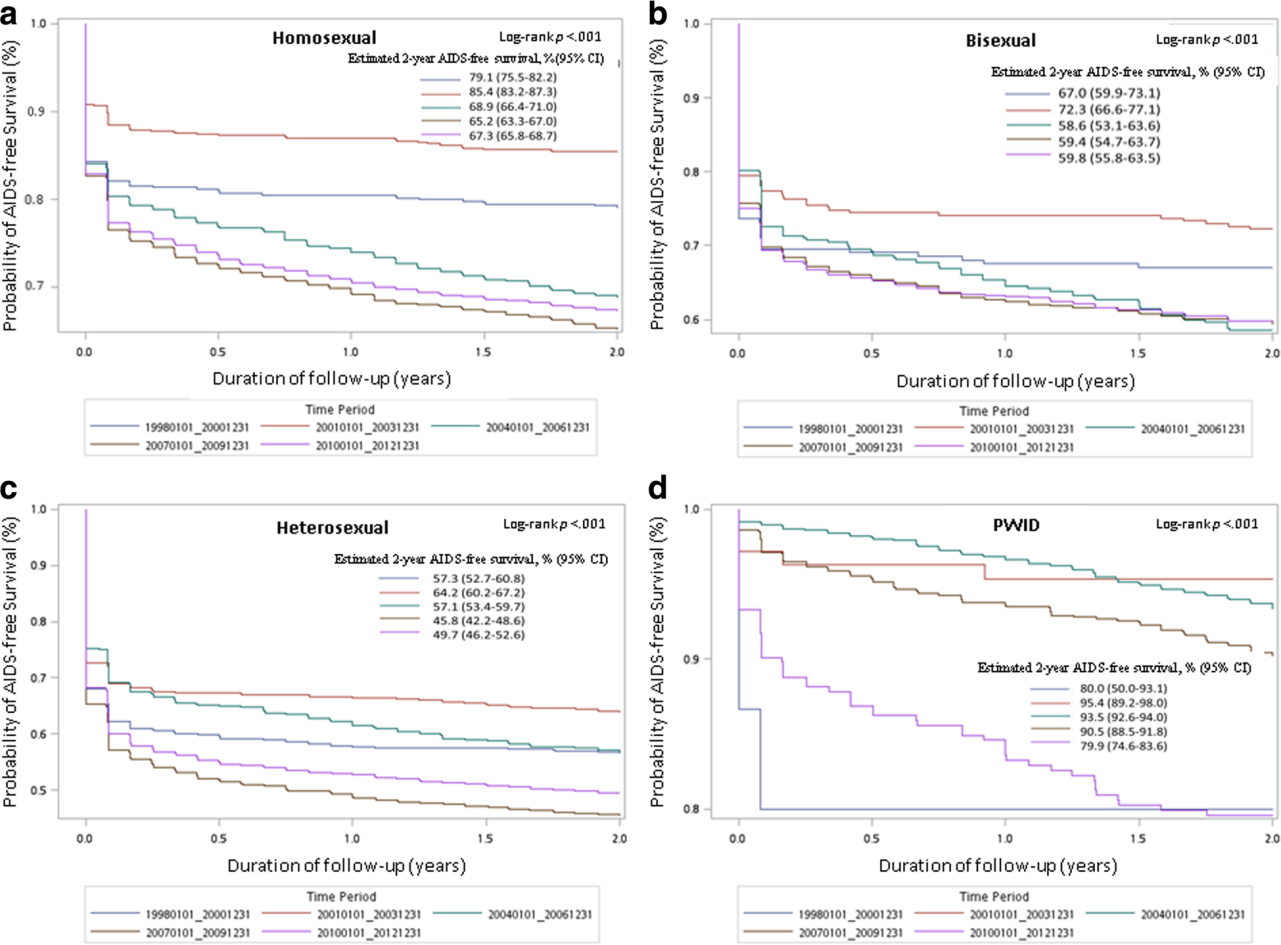
AIDS-free survival analysis stratified by five cohort periods in **a** homosexual, **b** bisexual, **c** heterosexual, and **d** PWID populations

**Fig. 3:**
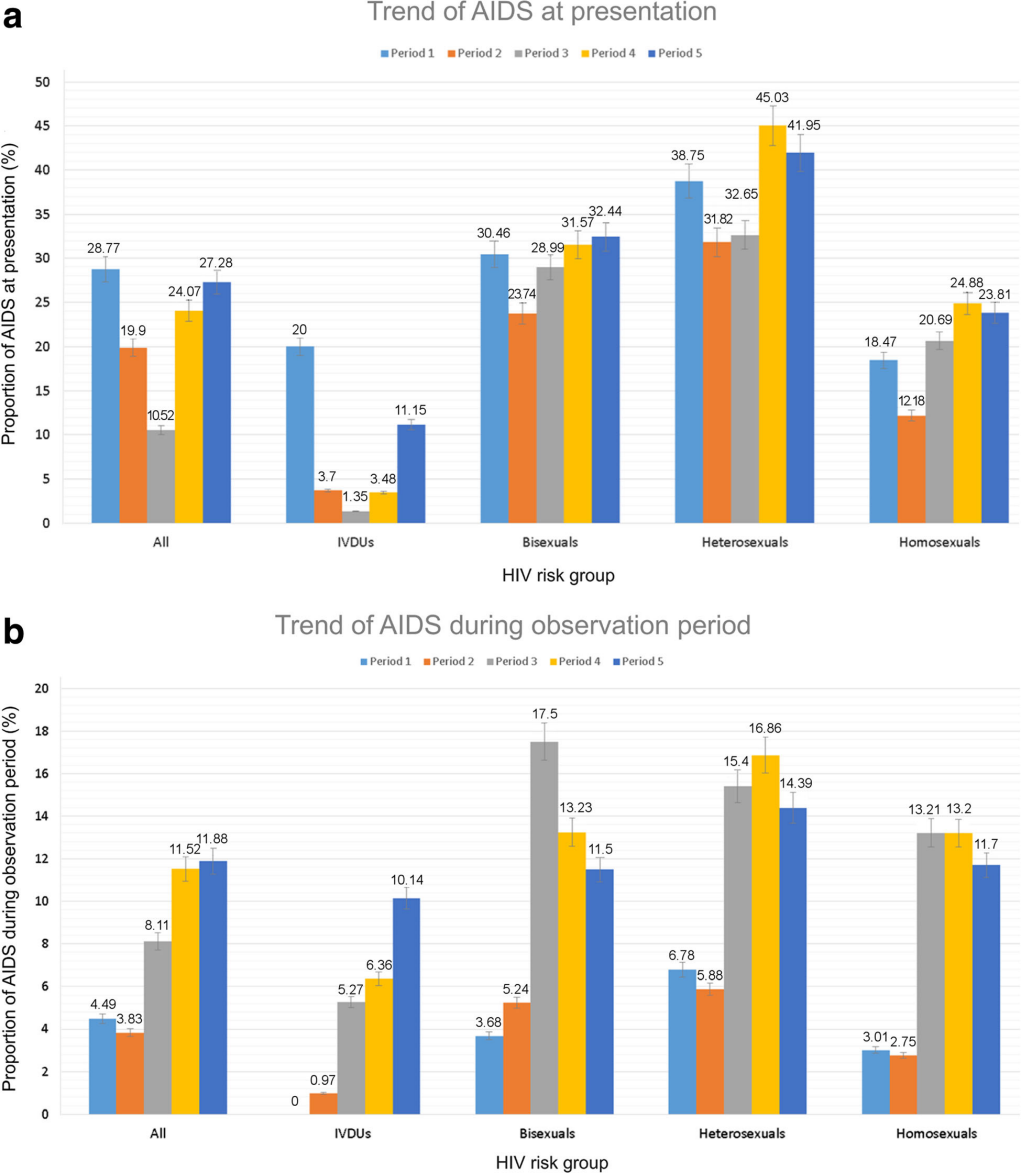
AIDS-incidence trends at presentation (**a**) and during the follow-up period (**b**) in HIV-transmission-route groups. **a** Overall, AIDS incidence at presentation declined from 28.8% in period 1 to a nadir of 10.5% in period 3 and then increased to 27.3% in period 5. After stratification by HIV-transmission route, however, AIDS incidence at presentation increased throughout the five cohort periods in the heterosexual (*P* for trend < 0.001), homosexual (*P* for trend < 0.001), and bisexual risk groups (*P* for trend = 0.648), whereas decreased to a nadir in period 3 and then increased to a peak in period 5 in in the PWID groups (*P* for trend < 0.001). **b** AIDS incidence during the follow-up period increased from 4.5% in period 1 to 11.9% in period 5. After stratification by HIV-transmission route, AIDS incidence during the follow-up period increased from period 1 to a peak in periods 3 and 4 and then declined in period 5 in heterosexual (*P* for trend < 0.001), homosexual (*P* for trend < 0.001), and bisexual risk groups (*P* for trend = 0.549), whereas in PWIDs it increased throughout the five cohort periods (*P* for trend < 0.001)

The probability of AIDS-free survival of various HIV-at-risk populations differed significantly throughout the five cohort periods (log-rank test, *P* < 0.001) (Fig. 2). AIDS incidence trends at presentation and during follow-up over the five cohort periods differed by HIV-transmission route (Fig. 3a and b). AIDS incidence at presentation increased in the sexual contact groups (*P* for trend < 0.001 for homosexuals/heterosexuals; *P* for trend = 0.648 for bisexuals), whereas that in PWIDs declined to a nadir in period 3 and then increased slightly in period 5 (*P* for trend < 0.001) (Fig. 3a). AIDS incidence during the follow-up period increased from period 1 to a peak in periods 3 or 4, and then declined slightly in period 5 in the sexual contact groups (*P* for trend < 0.001 for homosexuals/heterosexuals; *P* for trend = 0.549 for bisexuals), whereas it increased throughout the five cohort periods in the PWIDs (*P* for trend < 0.001) (Fig. 3b). Overall AIDS incidence at presentation and during the follow-up period was higher in the sexual contact population than in the PWIDs throughout the five cohort periods.

### Factors associated with AIDS incidence at presentation

Table 2 lists the factors associated with AIDS incidence at presentation. Logistic regression analysis revealed the following risk factors associated with AIDS incidence at presentation: older age (31–40 years, 41–50 years, and ≥ 51 years vs. ≤30 years, all *P* < 0.001); male (*P* < 0.001); HIV-transmission route (bisexuals, heterosexuals, homosexuals, and other contacts vs. PWIDs, all *P* < 0.001); cohort period (period 4 and period 5 vs. period 1, all *P* < 0.001); and region (all non-Taipei areas vs. Taipei area, all *P* < 0.01). High income level (vs. low income level, *P* < 0.01) and cohort Period 2 (vs. Period 1, *P* < 0.001) were factors that decreased AIDS incidence.

**Table 2:** Risk factors for AIDS incidence at presentation among patients newly diagnosed with HIV infection

	Number of patients, *n*	Number of AIDS, *n*	%	Univariable analysis, crude OR (95% CI)	Multivariable analysis, adjusted OR (95% CI)
Age group					
≤ 30	11,305	1445	12.8	1.0 (Reference)	1.0 (Reference)
31–40	6965	1594	22.9	2.03 (1.87–2.19)***	2.76 (2.53–3.01)***
41–50	2962	886	29.9	2.91 (2.65–3.21)***	4.19 (3.74–4.69)***
≥ 51	1433	646	45.1	5.60 (4.98–6.30)***	4.64 (4.00–5.37)***
Sex					
Female	1580	257	16.3	1.0 (Reference)	1.0 (Reference)
Male	21,085	4314	20.5	1.32 (1.15–1.52)***	1.39 (1.17–1.65)***
HIV-transmission route					
PWID	6646	154	2.3	1.0 (Reference)	1.0 (Reference)
Bisexual contact	1898	572	30.1	18.19 (15.08–21.93)***	20.28 (16.59–24.79)***
Heterosexual contact	4152	1592	38.3	26.22 (22.08–31.12)***	24.48 (20.39–29.39)***
Homosexual contact	9779	2147	22.0	11.86 (10.04–14.01)***	16.02 (13.35–19.22)***
Other^a^	190	106	55.8	53.19 (38.33–73.84)***	38.23 (26.94–54.24)***
Urbanization					
Rural	7384	1288	17.4	1.0 (Reference)	1.0 (Reference)
Urban	15,281	3283	21.5	1.30 (1.21–1.39)***	0.96 (0.88–1.05)
Income level, *n* (%)					
Low	13,785	2328	16.9	1.0 (Reference)	1.0 (Reference)
Intermediate	6421	1546	24.1	1.56 (1.45–1.68)***	0.99 (0.91–1.07)
High	2459	697	28.3	1.95 (1.76–2.15)***	0.85 (0.76–0.94)**
Cohort period					
Period 1	1373	395	28.8	1.0 (Reference)	1.0 (Reference)
Period 2	2262	451	19.9	0.62 (0.53–0.72)***	0.65 (0.55–0.77)***
Period 3	7774	819	10.5	0.29 (0.25–0.34)***	0.87 (0.75–1.01)
Period 4	5288	1274	24.1	0.79 (0.69–0.90)***	1.32 (1.15–1.53)***
Period 5	5968	1632	27.4	0.93 (0.82–1.06)	1.33 (1.15–1.53)***
HIV-diagnosis region					
Taipei area	7899	1656	21.0	1.0 (Reference)	1.0 (Reference)
Northern Taiwan	3212	608	18.9	0.88 (0.80–0.98)*	1.17 (1.05–1.32)**
Central Taiwan	4081	839	20.6	0.98 (0.89–1.07)	1.25 (1.13–1.39)***
Southern Taiwan	2687	525	19.5	0.92 (0.82–1.02)	1.26 (1.11–1.42)***
Kaoping area	4311	817	19.0	0.88 (0.80–0.97)**	1.18 (1.06–1.32)**
Eastern Taiwan	475	126	26.5	1.36 (1.10–1.69)**	1.27 (1.00–1.62)**
DM					
No	22,097	4362	19.7	1.0 (Reference)	1.0 (Reference)
Yes	568	202	35.6	2.26 (1.90–2.69)***	1.04 (0.84–1.29)
CKD					
No	22,358	4456	19.9	1.0 (Reference)	1.0 (Reference)
Yes	307	108	35.2	2.18 (1.72–2.76)***	1.28 (0.96–1.71)
CHF					
No	22,573	4522	20.0	1.0 (Reference)	1.0 (Reference)
Yes	92	42	45.7	3.50 (2.32–5.27)***	1.41 (0.85–2.36)
COPD					
No	22,158	4522	19.6	1.0 (Reference)	1.0 (Reference)
Yes	507	220	43.4	3.14 (2.62–3.75)***	1.63 (1.32–2.01)***
Cancer					
No	21,019	4061	19.3	1.0 (Reference)	1.0 (Reference)
Yes	1646	503	30.6	1.85 (1.65–2.06)***	1.15 (1.02–1.30)*
CVD					
No	22,408	4459	19.9	1.0 (Reference)	1.0 (Reference)
Yes	257	105	40.9	2.82 (2.20–3.62)***	1.33 (0.97–1.82)

Note. *P < 0.05; ** *P* < 0.01; *** *P* < 0.001

*Abbreviation*: *AIDS* acquired immunodeficiency syndrome; *CHF* chronic heart failure; *CI* confidence intervals; *CKD* chronic kidney disease; *COPD* chronic obstructive pulmonary disease; *CVD* cardiovascular disease; *DM* diabetes mellitus; *OR* odd ratios; *PWID* people who injected drugs

^a^Includes those exposed to blood products, mother-to-child transmission, and unknown exposures

### Factors associated with AIDS incidence during the follow-up period

Table 3 lists the factors associated with AIDS incidence during the follow-up period. On Cox regression analysis, the following factors were identified as risk factors associated with AIDS incidence during the follow-up period: older age (31–40 years, 41–50 years, and ≥ 51 years vs. ≤30 years, all *P* < 0.01); HIV-transmission route (bisexuals, heterosexuals, homosexuals, and other contact vs. PWIDs, all *P* < 0.001); cohort period (periods 3–5 vs. period 1, all *P* < 0.001); northern Taiwan (vs. Taipei area, *P* < 0.001), CKD (*P* < 0.05); and HAART during the follow-up period (*P* < 0.001). High income level (vs. low income level, *P* < 0.05) was protective factor.

**Table 3:** Risk factors for AIDS incidence during the follow-up among patients newly diagnosed with HIV infection

	Number of patients, *n*	Number of AIDS, *n*	%	Univariable analysis, crude HR (95% CI)	Multivariable analysis, adjusted HR (95% CI)
Age group					
≤ 30	9826	843	8.6	1.0 (Reference)	1.0 (Reference)
31–40	5312	442	8.3	0.97 (0.87–1.09)	1.17 (1.04–1.32)**
41–50	2028	227	11.2	1.35 (1.16–1.56)***	1.68 (1.44–1.96)***
≥ 51	737	127	17.2	2.19 (1.82–2.64)***	2.36 (1.90–2.92)***
Sex					
Female	1302	96	7.4	1.0 (Reference)	1.0 (Reference)
Male	16,601	1543	9.3	1.26 (1.03–1.55)*	1.16 (0.93–1.44)
HIV-transmission route					
PWID	6435	361	5.6	1.0 (Reference)	1.0 (Reference)
Bisexual contact	1322	148	11.2	2.05 (1.69–2.48)***	2.62 (2.11–3.24)***
Heterosexual contact	2496	312	12.5	2.33 (2.00–2.71)***	2.79 (2.35–3.31)***
Homosexual contact	7602	808	10.6	1.94 (1.71–2.19)***	2.58 (2.19–3.03)***
Other^a^	48	10	20.8	4.92 (2.62–9.22)***	4.90 (2.58–9.31)***
Urbanization					
Rural	6038	557	9.2	1.0 (Reference)	1.0 (Reference)
Urban	11,865	1082	9.1	0.99 (0.89–1.09)	0.93 (0.83–1.04)
Income level, n (%)					
Low	11,323	950	8.4	1.0 (Reference)	1.0 (Reference)
Intermediate	4833	508	10.5	1.26 (1.13–1.41)***	0.97 (0.86–1.08)
High	1747	181	10.4	1.24 (1.06–1.46)**	0.84 (0.71–0.99)*
Cohort period					
Period 1	957	43	4.5	1.0 (Reference)	1.0 (Reference)
Period 2	1777	68	3.8	0.85 (0.58–1.24)	0.82 (0.56–1.21)
Period 3	6890	559	8.1	1.84 (1.35–2.51)***	3.32 (2.42–4.56)***
Period 4	3976	458	11.5	2.67 (1.95–3.65)***	3.53 (2.57–4.84)***
Period 5	4303	511	11.9	2.76 (2.02–3.76)***	3.04 (2.22–4.17)***
Region					
Taipei area	6166	526	8.5	1.0 (Reference)	1.0 (Reference)
Northern Taiwan	2581	279	10.8	1.28 (1.11–1.48)***	1.41 (1.22–1.64)***
Central Taiwan	3209	275	8.6	1.00 (0.87–1.16)	1.06 (0.91–1.23)
Southern Taiwan	2137	197	9.2	1.09 (0.92–1.28)	1.17 (0.98–1.38)
Kaoping area	3466	324	9.4	1.10 (0.96–1.27)	1.14 (0.98–1.32)
Eastern Taiwan	344	38	11.1	1.30 (0.94–1.81)	1.16 (0.82–1.64)
DM					
No	17,556	1590	9.1	1.0 (Reference)	1.0 (Reference)
Yes	347	49	14.1	1.69 (1.27–2.25)***	1.08 (0.79–1.46)
CKD					
No	17,717	1610	9.1	1.0 (Reference)	1.0 (Reference)
Yes	186	29	15.6	1.89 (1.31–2.73)***	1.49 (1.02–2.17)*
CHF					
No	17,861	1630	9.1	1.0 (Reference)	1.0 (Reference)
Yes	42	9	21.4	2.82 (1.46–5.43)**	1.81 (0.92–3.58)
COPD					
No	17,626	1604	9.1	1.0 (Reference)	1.0 (Reference)
Yes	277	35	12.6	1.47 (1.05–2.05)*	1.00 (0.71–1.41)
Cancer					
No	16,788	1506	9.0	1.0 (Reference)	1.0 (Reference)
Yes	1115	133	11.9	1.38 (1.15–1.64)***	1.09 (0.91–1.31)
CVD					
No	17,765	1622	9.1	1.0 (Reference)	1.0 (Reference)
Yes	138	17	12.3	1.47 (0.91–2.36)	1.01 (0.62–1.65)
HAART during follow-up period					
No	5173	120	2.3	1.0 (Reference)	1.0 (Reference)
Yes	12,730	1519	11.9	1.43 (1.29–1.59)***	1.29 (1.15–1.44)***

Note. * *P* < 0.05; ** *P* < 0.001; *** *P* < 0.001

*Abbreviation*: *AIDS* acquired immunodeficiency syndrome, *CHF* chronic heart failure, *CI* confidence intervals, *CKD* chronic kidney disease, *COPD* chronic obstructive pulmonary disease, *CVD* cardiovascular disease, *DM* diabetes mellitus, *HAART* highly active antiretroviral therapy, *HR* hazard ratios, *PWID* people who injected drugs

^a^Includes those exposed to blood products, mother-to-child transmission, and unknown exposures

## Discussion

Although HAART has significantly altered the natural history of HIV infection in the cART era [1, 2], 27.4% patients still developed AIDS during the cohort periods, with a majority (73.6%) developing at HIV presentation. These findings indicate that in Taiwan, AIDS continues to pose a major threat to HIV patients, especially soon after diagnosis. Our study also revealed disparities in AIDS incidence trends in various HIV-at-risk populations. The disparities can be primarily explained by the different HIV-exposure-related sociodemographic factors and the influence of the adopted HIV prevention strategies; this knowledge will help prioritize resource reallocation for HIV diagnosis and the HIV-care continuum according to various targeted populations (Table 1, Fig. 3). The HIV epidemic in Taiwan is similar to that in other countries of the Asia-Pacific and North American regions [3, 4, 20, 21], where gbMSM are the predominant HIV transmission groups, and AIDS at presentation remains common. Therefore, this Taiwanese study’s findings should be of interest globally.

The late presentation (LP) of HIV is detrimental at both the individual [5–8] and general population levels [9, 10]. Different definitions have been adopted to indicate the LP of HIV, such as “a CD4 cell count of < 350 cells/*μ*L or AIDS-defining event at the first follow-up [3]” or “a CD4 cell count of < 200 cells/*μ*L or an AIDS-defining event within 3 months of HIV diagnosis [4]”. The lack of a standardized definition of LP makes it difficult to draw precise comparisons of the prevalence data reported from different studies. After using “a CD4 cell count of <200 cells/*μ*L or AIDS incidence in ≤3 months of HIV diagnosis” as a definition of LP (i.e., as the definition of AIDS incidence at presentation in the present study), the present study found a lower proportion of HIV patients with LP (20.2%) than that reported from other Asia-Pacific countries in the TREAT Asia HIV Observational Database (TAHOD) (72%) [4], China (27.3%) [3], and European countries [COHERE (33.2%)] [5]. The lower prevalence of LP in the present study compared with that in TAHOD may be attributable to a nationwide program of free anonymous VCT adopted in Taiwan since 1997 and enrollment of low-income countries in TAHOD, which is limited to health care access and health literacy. Even though the overall LP prevalence in Taiwan seems to be lower than that in other countries, HIV prevention and care in various HIV transmission groups remains a problem in Taiwan.

Similar to worldwide trends [3, 4, 20, 21], our study revealed that gbMSM has been emerging as the predominant HIV-transmission route, accounting for 78.2% new-HIV-infection cases in period 5. Furthermore, the trend of increasing LP among gbMSM in Taiwan showed no obvious signs of decline/reversal (Fig. 3). Therefore, reduction in the HIV epidemic and LP in gbMSM should be prioritized in Taiwan’s HIV prevention strategies. TCDC has adopted a wide range of HIV prevention programs among HIV-at-risk populations, especially the gbMSM population. A nationwide program of free anonymous VCT through a clinical service delivery model was initiated at several hospitals, community health centers, and nongovernmental organizations in 1997. The number of visitors who received VCT has increased steadily, and the percentage of new-HIV-infection cases through VCT increased from 9.0% in 2004 to 30.0% in 2010, after which it has plateaued [22]. In addition, TCDC also established gbMSM community health centers, employed services of online opinion leaders, and promoted VCT outreach services at saunas and pubs. Even though gbMSM have access to a broad range of free HIV services and organized community structures in Taiwan, the rising trend of LP in the gbMSM population indicates that HIV-at-risk gbMSM have been unaware/unwilling to receive HIV testing until late stages of HIV. Therefore, an integrated approach should be urgently employed to overcome HIV testing barriers in high-prevalence areas. These barriers include redressal of low risk perception [23], stigma and fear of discrimination [24], concerns about confidentiality [25], and possible structural barriers that contribute to poor HIV testing access in areas other than Taipei and among low-income population groups (Table 2). Taipei is the capital of Taiwan and has ample resources for HIV prevention and treatment [26].

Our study revealed that heterosexual contact is the second most common HIV-transmission route in Taiwan, accounting for 15.5% new-HIV-infection cases in period 5. Although the contribution of heterosexuals for HIV transmission is lower than that of gbMSMs, the findings reveal significantly higher LP prevalence among heterosexuals than in gbMSMs [adjusted OR (95% CI), 1.54 (1.40–1.70)] and an upward trend of LP among heterosexuals (*P* for trend < 0.001); this warrants a critical rethink of the HIV prevention strategies for both heterosexuals and gbMSMs in Taiwan. The finding of higher LP prevalence among heterosexuals in the present study is consistent with findings from other studies [5, 27], and may be attributable to the lower HIV testing rates among heterosexuals [28]. One explanation for this is the particular history of the HIV epidemic in Taiwan. Because HIV transmission in Taiwan has occurred predominantly within gbMSMs since 2008, HIV prevention and HIV-education strategies have long been shaped around gbMSMs. HIV prevention campaigns aimed at heterosexuals have either been lacking or were subsumed within the broader prevention campaigns focused primarily on other sexually transmitted infections. Other explanations include a lower risk perception and concerns regarding confidentiality [4, 29]. In addition, the rapid network and community-driven response to HIV prevention strategies among gbMSMs is not readily transferable to heterosexuals because of the wide diversity of sociodemographic variables (i.e., polyamory and commercial sex workers) of the HIV epidemic among the heterosexuals [20, 30]. Therefore, implementation of evidence-based population-specific prevention strategies is critical for achieving early HIV diagnosis in heterosexuals. In 2016, TCDC launched a program to distribute HIV home-based and self-testing kits to nongovernmental organizations, health stations, and to heterosexual and gbMSM communities. The high uptake and acceptance of these strategies among heterosexuals holds promise for increasing the uptake of HIV testing services among heterosexuals [31].

Taiwan experienced a major HIV epidemic among PWIDs during 2003–2008, and then a substantial reduction in HIV infection in PWIDs because TCDC launched harm-reduction programs in August 2005 [14]. Consequently, only 3.2% (77/2406) of new-HIV-infection cases were attributed to PWIDs in Taiwan in 2016 [14, 32]. In contrast to some Asian and European studies [4, 5], PWIDs were not identified as a major LP risk group in our study (Table 2). A possible cause of the discrepancy may be the predominance of the HIV CRF07_BC strain among PWIDs in Taiwan, which is characterized by slow immunological progression [33, 34]; another reason could be the legal implementation of an active surveillance for prison inmates since 1991 with resultant early diagnoses of HIV in PWIDs [15, 35].

Consistent with relevant studies, the present study also identified old age as an LP risk factor [4, 36] (Table 2). These results are likely attributable to changes in risk-related behavior (i.e., decreased condom use) and lower HIV-risk perception among older people, which resulted in a higher proportion of diagnosis during hospitalization [37]. Moreover, primary physicians may not consider the possibility of HIV infection among the older population because of masking by multiple comorbidities [38], variations in HIV symptom manifestation [39], and underestimation of HIV-risk transmission behaviors [40]. Effective HIV prevention education for early HIV diagnosis in older populations rests in part on countering the misconceptions about HIV transmission risk and on enhancing the awareness of healthcare providers.

Mathematical models suggest that universal HIV testing followed by immediate treatment can decrease HIV incidence at the population level [41]. Although active surveillance programs among specific populations in Taiwan have been consecutively adopted since 1988, anonymous VCT has been a strategic priority for HIV testing among the sexual contact population since 1997. However, testing according to the perceived HIV transmission risk and willingness to undergo HIV testing among the sexual contact population likely contributes to the increasing LP prevalence in HIV-at-risk populations (Fig. 3a). HIV-screening implementation as part of routine care or implementation of opt-out testing in both healthcare and nonhealthcare settings may help increase HIV testing rates, destigmatize it, and improve healthcare access for new-HIV-infection cases [42, 43]. However, routine HIV-screening approaches in HIV-at-risk populations without permission, except in specific situations, are prohibited by law in Taiwan. Therefore, an integrated approach to scale-up current VCT strategies tailored to HIV-at-risk populations is urgently required to increase HIV testing coverage among the sexual contact and older populations and among those who reside outside of the Taipei area (Table 2). This integrated approach should include education to increase HIV-risk perception, eliminate stigma and discrimination, and adopt a flexible approach to HIV testing, including peer-based [44] or home-based HIV testing services [31], and indicator-guided HIV testing by healthcare providers [45, 46].

Another valuable finding of the present study is the disparity in AIDS incidence trends during the follow-up period among the various HIV-at-risk populations in the five cohort periods (Fig. 3). The higher AIDS prevalence during follow-up in the sexual contact population than among PWIDs in Taiwan may be attributable to the higher LP prevalence in the sexual contact population (Table 2) which may result from low risk perception, stigma and discrimination, concerns about confidentiality, and uneven distribution of resource for HIV prevention as mentioned above, and be intrinsically linked to HIV-1 genetic diversity in Taiwan. Serotype B is the predominant strain in the sexual contact groups, whereas CRF_07 BC is the major strain in PWIDs [33], which is characterized by slower immunological progression compared with those in serotype B [33, 34]. Several crucial strategies have been implemented to increase the HIV-care continuum in Taiwan, including access to free HAART since 1997, initiation of HAART at higher CD4-count thresholds (CD4 < 200 cells/mm^3^ in 2006, < 350 cells/mm^3^ in 2010, < 500 cells/mm^3^ in 2013, and in all patients, regardless of CD4 count, in 2016), an HIV case management program since 2007, and the employment of a single tablet regimen (STR) in HAART since 2016. We believe that AIDS prevalence during the follow-up period can be decreased substantially with HAART initiation for all patients and with STR implementation.

However, unlike in the sexual contact population, the rapid increase in AIDS incidence trends during the follow-up period among PWIDs (*P* for trend < 0.001) should be cautiously monitored (Fig. 3b). This increasing trend among PWIDs may be attributable to suboptimal adherence to HAART among prison inmates [47], a higher rate of HIV-care discontinuity [48–50], and deferral of HAART by physicians [51]. Considering the sharp decrease in adherence to ART after release of incarcerated PWIDs [52], treatment of addiction and HIV infection at imprisonment and after release is critical for improving the continuity of post-release care [53]. We also identified chronic kidney disease as a risk factor for AIDS during the follow-up (Table 3). These results are likely attributable to the defective immune system through both immune activation and immune suppression [54], which might result in the reactivation of *Mycobacterium tuberculosis* or other AIDS-defining opportunistic infections [55–57]. In summary, given the diversity of care needs of the various HIV-at-risk populations, identification of the modifiable factors associated with AIDS incidence after linking to the HIV care is vital to better customize the HIV-care strategies for HIV-at-risk populations in Taiwan.

The major strength of the present study was its nationwide scope and population-based design combined with the long and nearly complete follow-up. The population-based design minimized selection and referral biases. The findings are critical for public health policies with regard to AIDS control, and may be generalized to Asia-Pacific and North American countries, which face similar HIV epidemics. However, the study had some limitations. First, the data used was limited to data reported to TCDC and NHIRD by primary physicians. Second, whether unmasking of the type of opportunistic illnesses after HAART contributed to AIDS incidence after HAART was not ascertained.

## Conclusions

The results revealed that 27.4% patients developed AIDS during the cohort periods, with a majority developing at presentation, which reinforces the importance of identifying HIV infections as early as possible. The disparities in AIDS incidence trends at presentation and during follow-up in various HIV-at-risk populations can be explained by different sociodemographic variables associated with HIV exposure and the influence of the adopted HIV prevention strategies. These results suggest an urgent need for more active offering of HIV testing among sexual contact groups, with different policies tailored for gbMSMs (increased risk perception, removal of stigma and discrimination, protection of confidentiality, and elimination of structural barrier to HIV testing) and heterosexuals (implement of HIV home-based and self-testing kits). After linking to the HIV care, differentiated care for specific populations is required to better customize HIV-care strategies, especially for PWIDs (increased adherence to HIV care and HAART, especially after release, and increase willing of prescription of HAART by physicians).

## Abbreviations

AIDS: Acquired immunodeficiency syndrome
CHF: Chronic heart failure
CKD: Chronic kidney disease
COPD: Chronic obstructive pulmonary disease
CVD: Cardiovascular disease
DM: Diabetes mellitus
gbMSM: Gay, bisexual, and men who have sex with men
HAART: Highly active antiretroviral therapy
HR: Hazard ratio
IQR: Interquartile range
LP: Late presentation
NDSS: Notifiable Diseases Surveillance System
NHIRD: National Health Insurance Research Database
NTD: New Taiwan Dollars
OR: Odds ratio
PWID: Intravenous drug user
SD: Standard deviation
TCDC: Taiwan Centers for Disease Control
VCT: Voluntary counseling and testing
